# Aging Changes Effective Connectivity of Motor Networks During Motor Execution and Motor Imagery

**DOI:** 10.3389/fnagi.2019.00312

**Published:** 2019-11-21

**Authors:** Li Wang, Ye Zhang, Jingna Zhang, Linqiong Sang, Pengyue Li, Rubing Yan, Mingguo Qiu, Chen Liu

**Affiliations:** ^1^Department of Medical Imaging, College of Biomedical Engineering, Army Medical University, Chongqing, China; ^2^Department of Rehabilitation, Southwest Hospital, Army Medical University, Chongqing, China; ^3^Department of Radiology, Southwest Hospital, Army Medical University, Chongqing, China

**Keywords:** motor network, motor execution, motor imagery, effective connectivity, Granger causality analysis

## Abstract

Age-related neurodegenerative and neurochemical changes are considered to be the basis for the decline of motor function; however, the change of effective connections in cortical motor networks that come with aging remains unclear. Here, we investigated the age-related changes of the dynamic interaction between cortical motor regions. Twenty young subjects and 20 older subjects underwent both right hand motor execution (ME) and right hand motor imagery (MI) tasks by using functional magnetic resonance imaging. Conditional Granger causality analysis (CGCA) was used to compare young and older adults’ effective connectivity among regions of the motor network during the tasks. The more effective connections among motor regions in older adults were found during ME; however, effective within-domain hemisphere connections were reduced, and the blood oxygenation level dependent (BOLD) signal was significantly delayed in older adults during MI. Supplementary motor area (SMA) had a significantly higher In+Out degree within the network during ME and MI in older adults. Our results revealed a dynamic interaction within the motor network altered with aging during ME and MI, which suggested that the interaction with cortical motor neurons caused by the mental task was more difficult with aging. The age-related effects on the motor cortical network provide a new insight into our understanding of neurodegeneration in older individuals.

## Introduction

Age-related neurodegenerative and neurochemical changes have been believed to impair motor performance and cognitive functions. Many fMRI studies have investigated motor functional changes with aging. During the visual paced “button” motor task, the older subjects show a greater degree of activation in the contralateral sensorimotor cortex, lateral premotor area, and SMA as well as in other activation areas compared to young subjects; the ipsilateral sensorimotor cortex is not obviously activated in young subjects (Mattay et al., 2002). There is a greater extent of activation in the ipsilateral motor cortex during unimanual movement in older subjects compared to younger subjects (Carp et al., 2011). Most of these results are explained as a compensatory mechanism in the motor system to compensate for motor performance decline with aging (Mattay et al., 2002; Talelli et al., 2008a; Seidler et al., 2010). In contrast, many studies focus on how to delay the age-related decrease in motor performance, and some researchers have suggested that mental training through motor imagery (MI) may be beneficial to improving motor function (Jeannerod and Decety, 1995; Jeannerod, 2001).

Motor imagery is a dynamic cognitive process during which a movement is mentally simulated without being actually executed (Jeannerod and Decety, 1995; Saimpont et al., 2015). Many studies have reported that patients over 60 years of age in the field of neurorehabilitation can potentially benefit from MI training programs (Jeannerod and Decety, 1995; Jeannerod, 2001). The theoretical basis of MI for motor rehabilitation is that MI shares the same neural substances with motor execution (ME) and could promote the interaction between the brain area (Jeannerod and Decety, 1995). Reciprocal interactions between SMA and the bilateral dorsal premotor area and between the SMA and the primary motor cortex (M1) have been found during MI. However, the interactions among these cortical motor regions have mainly been studied in young adults (Chen et al., 2009), and it was not clear whether the interactions changed with aging during MI.

The popular techniques to investigate direct interactions include structural equation modeling (SEM) (Gates et al., 2011; Kiyama et al., 2014) and dynamic causal modeling (DCM) (Raichle and Snyder, 2007). These lagged effects were not considered in SEM models of fMRI, and the DCM assumes an influence between the two regions of interests (ROI) in advance, which may lead to incorrect results because of the erroneous estimation of the influence on the connected brain area. Granger causality modeling may be an appropriate approach for studying the directional interactions between these brain regions and reveal complex temporal and spatial dynamics underlying a variety of motor and cognitive processes (Goebel et al., 2003; Gao et al., 2008a; Liao et al., 2009). An important extension to Granger’s original definition is the Conditional Granger causality analysis (CGCA), which takes into account that the multivariate time series (the causal relation between any two of the series) could be direct, mediated by third series, or a combination of the two (Gao et al., 2011; Zhou et al., 2011).

Therefore, with the aim of obtaining a clear picture of the networks of interaction among cortical motor regions, we employed the CGCA method to investigate age-related changes of effective connectivity in the cortical motor network. We have increased activity in motor regions during ME and decreased activity during MI in our previous work (Wang L. et al., 2014). Thus, in the current study, we hypothesized that (1) the interaction between motor regions would increase with aging during ME and (2) the interaction between motor regions would decrease with aging during MI.

## Materials and Methods

### Subjects

Forty healthy right-handed subjects were recruited, including 20 elderly and 20 young. All subjects were evaluated for MI performance and cognitive function by using the Movement Imagery Questionnaire-Revised (MIQ-RS) (Ding et al., 2000; Butler et al., 2012) and Mini-Mental State Examination (MMSE). Each subject gave informed consent and experimental protocols were obtained from the Ethics Committee of the Third Military Medical University. The study was performed in accordance with the approved guidelines.

#### Scanning Procedures

Imaging was carried out on a 3.0-T Siemens Medical MRI scanner (Trio, Erlangen, Germany). A gradient-recalled echo planar imaging (EPI) sequence was employed for fMRI scanning using the following parameters: TR = 2000 ms; TE = 30 ms; FOV = 220 mm; matrix = 64 × 64; slice thickness = 3 mm with 1 mm gap, 27 slices; and flip angle = 90°. High-resolution T1-weighted structural images were obtained using the 3D MP-RAGE pulse sequence with the following parameters: TR = 1900 ms, TE = 2.52 ms, flip angle = 15°, matrix = 256 × 256, FOV = 240 mm, thickness = 1.0 mm, 176 contiguous axial slices with voxel size = 3.47×3.47×4*mm*^3^. The subjects lay in the scanner and remained quiet during the scan.

### Experimental Procedure

**FIGURE 1:**
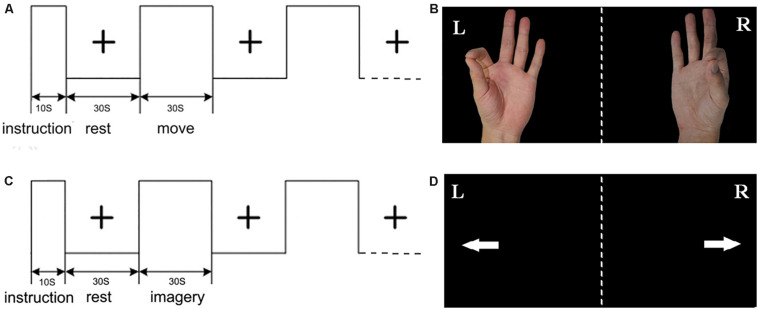
Experimental design: **(A)** the block of motor execution (ME); **(B)** picture of right/left hand finger during ME; **(C)** the block of motor imagery (MI); **(D)** picture of right/left arrow during MI; each run included a 10 s instruction introduction period and five blocks, and each block included 30 s of rest and 30 s of task.

The present study is an extension of our previous studies (Wang L. et al., 2014). The experimental procedure primarily included right hand index finger-thumb oppositional movements and imagining of the same movements without actually performing them. As shown in Figure 1, for the ME, the picture of left/right hand finger movement at a frequency of 1 Hz (1 s, including the relaxation and pinch) was presented on the screen. For the MI, the picture of left/right arrow was presented on the screen. For the rest, the black central cross picture was presented on the screen, which prompted the subjects to place their hands on the sides of the body and to breathe calmly (Wang L. et al., 2014, Wang et al., 2016). The fMRI was conducted with an established block design for each run. Each run included a 10 s instruction introduction period and five blocks, and each block included 30 s of rest and 30 s of tasks. All visual stimuli presentations and behavioral recordings were implemented using the E-prime software. Subjects wore the fiber optic gloves (Fifth Dimension Technologies, Pretoria, South Africa) in the whole experiment. Data gloves were connected to the computer. Each subject’s movement of their two hands in real time within the nuclear magnetic resonance instrument was observed by the computer. Prior to the fMRI experiment, participants were trained for approximately 1 h two times.

### Data Preprocessing

The statistical parameter mapping software was used to preprocess the experimental data (SPM8, https://www.fil.ion.ucl.ac.uk/spm/). All original images were reoriented and realigned. The subjects whose head movement exceeded 2 mm or rotation exceeded 2 degrees were excluded from subsequent analysis. The functional images are rearranged and normalized into standard stereo positioning space. The voxels were re-segmented into 3 mm × 3 mm × 3 mm voxels and smoothed with 6 × 6 × 6 full width at the half maximum core. General linear model analysis was applied to the data from each subject following high-frequency filtering and global scaling with SPM8. Next, the task-related t-contrast images (ME > 0 and MI > 0) were calculated for each subject using the t-statistics. Covariate statistical analysis was performed with 6 head motion parameters, white matter signals and cerebrospinal fluid signals as covariates. A one-sample *t*-test was then performed across subjects based on the contrast images between the ME/MI condition and the rest condition. The SPM {t} s were thresholded at *P* < 0.01 (false discovery rate (FDR) correction). The results of these individuals and groups would be used for subsequent analysis.

#### Blind Hemodynamic Response Functions (HRF) Deconvolution

After data preprocessing, we detected spontaneous and evoked point events in five task blocks and the resting conditions, respectively, by using the point process analysis. BOLD fluctuations of relatively large amplitude (>1SD) were collected and the onsets of neural event were saved for HRF reconstruction. The HRF in each voxel was obtained by matching BOLD signal with the canonical HRF and its time derivative. The neural level signals were recovered by using Wiener deconvolution (https://users.ugent.be/∼dmarinaz/HRF_deconvolution.html) (Wu et al., 2013; Wang Y.F. et al., 2014, 2015; Wang Y. et al., 2015).

### Identification of the Regions of Interest

Besides the premotor cortices (PMC), SMA and M1, Cerebellum, basal ganglia, and other regions also become activated. These regions might play an important role in the MI and ME. In this study, our aims are to investigate the interaction of cortical regions, so we choose ROI within the motor cortex. After HRF deconvolution, the SMA, bilateral PMC and M1 were selected as ROIs for the comparisons of functional brain networks between young and older subjects during ME and MI. Due to the anatomical variation and functional alignments, we defined the ROIs by the individual’s and group’s t-contrast maps (Jiang et al., 2004; Hattori et al., 2009). First, the 10-mm-radius spheres around the peak voxel of the brain regions were defined according to the group’s SPM {t} maps. The MNI coordinates of the center of each sphere are in Table 1. Second, using a given group ROI as a mask, the individual peak voxel was picked up with the maximum *t*-value within this mask. Then, a sphere with a radius of 6 mm was defined around the highest positive *t*-value voxel as an individual ROI (Xu et al., 2014; Wang Y. et al., 2015). For each subject and each stimulus condition, mean time series in each ROI were obtained by averaging the functional MRI time series across all voxels in the ROI. To detrend and demean the mean time series, the best-fitting line was subtracted from each time series, and the temporal mean value was removed from each time series to provide a “zero-mean” value. These time series formed the basis for CGCA.

**TABLE 1:** The MNI coordinates of the center of the ROI.

**Anatomic site**	**ME**				**MI**			
	**Side**	***x***	***y***	***z***	**Side**	***x***	***y***	***z***
**Older**								
M1	L	−36	−24	63	L	−51	−9	45
M1	R	57	−15	27	R	51	−9	48
PMC	L	−36	−21	63	L	−30	−6	60
PMC	R	42	−3	45	R	48	−3	51
SMA		6	3	57		−6	0	57
**Young**								
M1	L	−36	−24	51	L	−48	−9	48
M1	R	60	−12	30	R	60	−15	27
PMC	L	−39	−18	63	L	−42	−6	42
PMC	R	51	3	48	R	51	3	45
SMA		−3	−3	57		−6	9	45

### Causal Influences Among ROIs

Granger causality is a statistical hypothesis test used to determine whether one time series can help predict another time series. Conditional Granger causality is an extension of Granger causality. If there were 3 variables in the Granger causality analysis, the causal relation between any two of the time series of the system includes (1) direct interaction effects; (2) indirect interaction effects mediated by the third one; and (3) combination of both direct and indirect.

The joint vector autoregressive representation for *x*_*t*_ and *y*_*t*_ was:

$$x_{t}=\sum_{i=1}^{\infty }a_{i\,i}\,x_{t-i}+\sum_{i=1}^{\infty }c_{i\,i}\,y_{t-i}+\varepsilon_{1\,t}$$

$$y_{t}=\sum_{i=1}^{\infty }b_{i\,i}\,y_{t-i}+\sum_{i=1}^{\infty }d_{i\,i}\,x_{t-i}+\varepsilon_{1\,t}$$

If there were three time series*x*_*t*_,*y*_*t*_,*z*_*t*_, obtaining Granger causality by vector autoregressive modeling

$$x_{t}=\sum_{i=1}^{\infty }a_{2\,i}\,x_{t-i}+\sum_{i=1}^{\infty }b_{2\,i}\,y_{t-i}+\sum_{i=1}^{\infty }c_{2\,i}\,z_{t-i}+\varepsilon_{3\,t}$$

$$y_{t}=\sum_{i=1}^{\infty }d_{2\,i}\,x_{t-i}+\sum_{i=1}^{\infty }e_{2\,i}\,y_{t-i}+\sum_{i=1}^{\infty }f_{2\,i}\,z_{t-i}+\varepsilon_{4\,t}$$

$$z_{t}=\sum_{i=1}^{\infty }g_{2\,i}\,x_{t-i}+\sum_{i=1}^{\infty }h_{2\,i}\,y_{t-i}+\sum_{i=1}^{\infty }k_{2\,i}\,z_{t-i}+\varepsilon_{5\,t}$$

The actual state *x* at time point *t* is derived from its past states with additional input from a Gaussian white noise process ε(t).

Conditional Granger Causality from *x* to *y* conditioned on z is:

$$F_{y\to x|z}\equiv \ln\left(\frac{v\,a\,r\,\left(\varepsilon_{1\,t}\right)}{v\,a\,r\,\left(\varepsilon_{3\,t}\right)}\right)>0$$

When the G-causal effect from *y* to *x* is entirely caused (mediated) by *z*, the *b*_2*t*_≡0 and var(ε_1*t*_) = var(ε_3*t*_); that is why *F*_*y*→*x*|*z*_≡0, i.e., the past values of *y* cannot improve the prediction of the current value of *x* conditioned on *z*. When there is a direct effect from *y* to*x* over the one caused by the mediation of *z*, the inclusion of past values of *y* in addition to that of *x* and *z* improves the prediction of the current value of *x*, hence *var*(ε_1*t*_) > *var*(ε_3*t*_),*F*_*x*→*y*|*z*_ > 0. The Granger causality was applied to the fMRI data of task; the vector time series of one activated ROI is correlated with *x*_*t*_ and another one ROI with *y*_*t*_. The residual of the ROIs’ time series is correlated with *z*_*t*_.

The Granger Causal Connectivity Toolbox (Seth, 2010) estimated the Granger causality in Matlab (Natick, MA, United States). First, all variables were checked covariance stationary by using the Augmented Dickey–Fuller (ADF) test. Second, linear trends and the temporal mean from each observation of the time series were removed. Three, the optimal mode order 1 was determined by the application of Bayesian Information Criterion. Then, the Granger causality was calculated using the Bonferroni Criterion, and the statistical significance of Granger causality value was estimated under the *F*-test; subsequently, the conditional Granger causality was performed and eliminated the mediated interactions. To assess the between-group differences of Granger causality estimate results, we used Wilcoxon signed rank test for non-parametric statistical analyses. The multiple linear regression analyses were used for permutation test before excluding the effects of confounding factors, including age, gender, and MI scores. These connections were significant, and the acceptable FDR of all connections was *q* < 0.05. In- and Out-degree was calculated and compared between groups.

### In- and Out-Degree Calculated and Compared Between Groups

Degree is an important indicator of graph theory of network. It defines the number of edges a node has to other nodes, which is a measure of node importance in the network. A greater degree indicates that the node is more important in the network. Out-degree refers to the number of edges from the node to other nodes, and In-degree refers to the number of the edges from other nodes to the node. Because the nodes (five ROI) are relatively small in this study, we combined the In- and Out-degrees of ROI together for analysis. We calculated the average of In- and Out-degree across all subjects in each group during performance of each task.

### Behavioral Data

For the older group, the mean age was 62.1 ± 7.0, the mean imagination score was 32.7 ± 3.3, and the mean MMSE score was 28.1 ± 1.4. For the young group, the mean age was 21.6 ± 0.9 years, the mean imagination score was 35.6 ± 2.7, and the MMSE score was 29.7 ± 0.7. Comparing the behavioral data, the scores of MMSE and MIQ-RS did not reveal significant differences between the groups.

### Brain Activation

**FIGURE 2:**
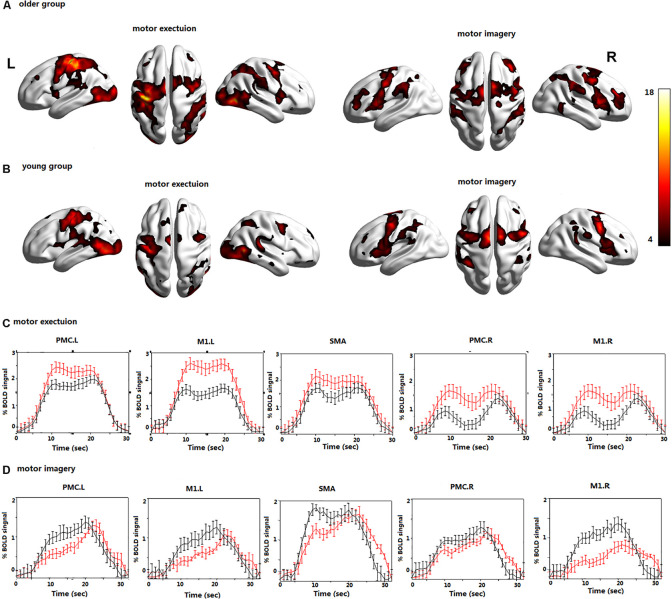
The brain activations during motor execution (ME) and motor imagery (MI) in **(A)** the older group; **(B)** the young group; the BOLD signals averaged across trials and subjects and presented as the mean ± standard error for the older (red) and young group (black) during **(C)** ME; **(D)** MI; all voxels are significant at *P* < 0.01. FDR corrected for the whole brain. Left premotor cortex (PMC.L); right premotor cortex (PMC.R); supplementary motor area (SMA); left primary motor cortex (M1.L); and right primary motor cortex (M1.R).

In the young group, the contralateral PMC, SMA, and M1 were significantly activated during ME. The bilateral PMC, SMA, and M1 in the older group were significantly activated during ME, and the activation intensity was stronger than that of young adults (Figure 2).

In the young group, the bilateral PMC, SMA, and M1 were significantly activated during MI. In the older group, the bilateral PMC, SMA, and M1 were activated during MI, and the activation intensity was significantly lower than that of young adults (Figure 2).

The average time course of the ROIs across all blocks and all subjects revealed clear differences in the BOLD signals during the MI and ME in both groups (Figure 2). The BOLD response was significantly higher in the older group than in the young group during ME, and the opposite results were observed during MI. The BOLD response is consistent with the previous activation of brain regions. In addition, the time series lagged in activation of the ROIs during MI in the older group; however, there was no obvious time lag in the activation of the ROIs during ME.

### Causal Connectivity Analyses

There were many reciprocal interactions within the motor network during ME in both groups (Figure 2 and Table 2). In the older group, there were many strong between-hemisphere connections; however, no such phenomenon was observed in the young group. The within-hemisphere connections were stronger in the older group than in the young group during ME. These connections were mainly involved in the influence of the right PMC on the left PMC, left M1, and the right M1.

**TABLE 2:** The value of conditional Granger causality in the older group during ME and MI.

	**PMC.L**		**PMC.R**		**M1.L**		**M1.R**		**SMA**	
	**ME**	**MI**	**ME**	**MI**	**ME**	**MI**	**ME**	**MI**	**ME**	**MI**
PMC.L	/	/	0.027	0.030	0.054	0.033	0.067	0.035	0.045	/
PMC.R	0.031	/	/	/		0.045	0.031	/	/	/
M1.L	0.010	0.019	/	/	/	/	0.040	0.043	0.028/	0.021
M1.R	/	/	0.018	0.028	0.048	/	/	/	/	/
SMA	0.022	0.047	0.024	0.043	0.032	0.043	0.058	/	/	/

**FIGURE 3:**
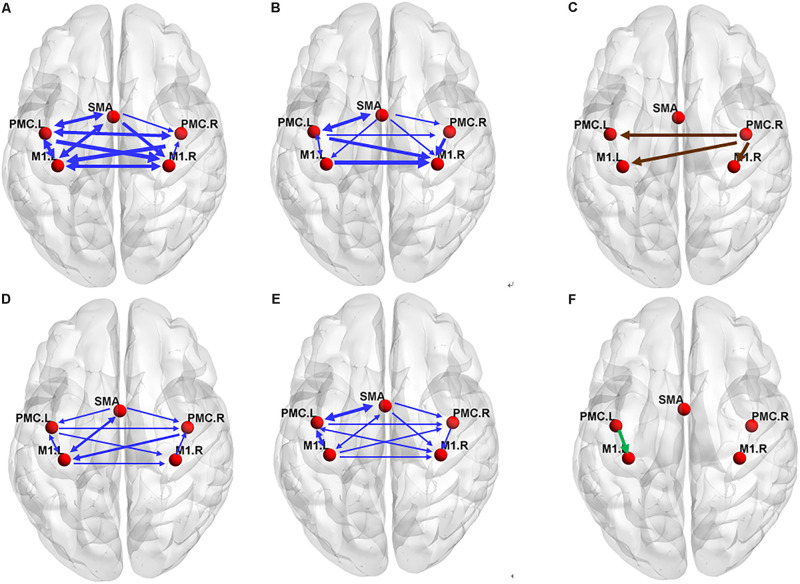
The effective connectivity networks during motor execution (ME) in the older **(A)** and young group **(B)** and two-sample statistical results **(C)**. Effective connectivity networks during motor imagery (MI) in the older **(D)** and young group **(E)** and two-sample statistical results **(F)**. The thickness of the lines is proportional to the connection strength. The connections were tested with non-parametric statistical analyses using Wilcoxon signed rank test and corrected for multiple comparisons (FDR, *p* < 0.05).

In the older group, there was a decreased interaction during MI, and the connectivity strength dramatically decreased. The reduced within-hemisphere connections were a causal influence from left M1 to left PMC (Figure 3 and Table 3).

**TABLE 3:** The value of conditional Granger causality in the young group during ME and MI.

	**PMC.L**		**PMC.R**		**M1.L**		**M1.R**		**SMA**	
	**ME**	**MI**	**ME**	**MI**	**ME**	**MI**	**ME**	**MI**	**ME**	**MI**
PMC.L	/	/	0.045	0.026	0.035	0.028	0.022	0.035	0.018	0.026
PMC.R	/	/	/	/	/	/	0.054	0.024	/	/
M1.L	0.027	0.020		0.019	/	/	0.060	0.028	/	0.035
M1.R	/	/	/	/	/	/	/	/	/	/
SMA	0.020	0.020	0.040	0.032	0.017	0.012	0.030	0.031	/	/

**FIGURE 4:**
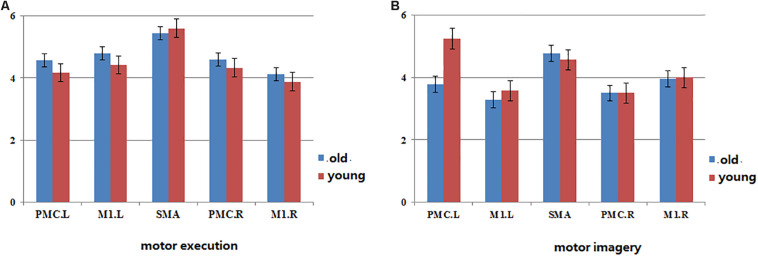
The map of statistical analysis of In+ut degree in each ROI in the groups. Blue bar indicates the average of In+Out degree across all subjects in old group, red bar indicates the average of In+Out degree across all subjects in young group. **(A)** The In+Out degree in motor network during motor execution in young and old group. **(B)** The In+Out degree in motor network during motor imagery in young and old group.

The In+Out degree of SMA was significantly higher (*P* < 0.01) within the motor network during ME in both groups; however, the In+Out degree of left PMC was significantly higher (*P* < 0.01) within the motor network during MI in the young group. The In+Out degree of SMA was significantly higher (*P* < 0.01) in the older group (Figure 4).

## Discussion

In this study, we found that the interactions between the cortical motor regions increased in older adults during ME and decreased during MI by using CGCA. Our approach was expected to reveal the cause of the motor network during ME and MI and to supply information that would enable a deeper understanding of age-related neurodegenerative and neurochemical motor network changes.

### Brain Activations and BOLD Signals

The bilateral SMA, PMC, and M1 were dramatically activated in both groups during ME; however, more brain regions and stronger activated intensity were activated in the older group. This was consistent with greater brain activation of many areas of the motor system in older adults, particularly when they implemented more complex movement tasks (Calautti et al., 2001; Hutchinson et al., 2002; Mattay et al., 2002; Seidler et al., 2010). This may be related to the compensatory recruitment of motor cortical units in older subjects (Talelli et al., 2008b; Seidler et al., 2010). In addition, we found many ipsilateral motor cortexes in the older group that were activated during ME. These may be related to aging, which leads to a reduction in interhemispheric inhibition and increased activation in the ipsilateral hemisphere (Talelli et al., 2008b; Seidler et al., 2015). We detected SMA, bilateral PMC, and M1 dramatic activation in both groups during MI, thus confirming that the actual execution and its mental representation involve the activation of similar brain areas (Jeannerod, 2001). Compared with the older group, there was stronger activation of motor regions in the young group, implying that with aging, that activation in the motor regions becomes more difficult during MI.

Interestingly, the BOLD signal of motor regions lagged, and in addition intensities were lower in the older group during MI. The delayed response of cortical motor regions in the older group during MI is more likely to be caused by neurodegeneration. Age-related neurodegenerative and neurochemical changes are considered to be the basis for the decline of motor and cognitive function (Wenk et al., 1989; Newson and Kemps, 2005). Some studies found that older adults were significantly slower than young adults in imagining Fitts-like tasks (Skoura et al., 2005; Personnier et al., 2010), especially when they imagined movement with their non-dominant hand (Skoura et al., 2008). In this study, the blood oxygen response induced by MI was significantly delayed in older adults, which further indicated that the activation of brain areas caused by mental activity was more difficult with aging. The BOLD signal did not obviously lag in the older group during ME, which may be related to simple motor performance and requires further research.

### Effective Connections During ME

Regarding the ME, our results revealed a high level of consistency in the connections between the groups in terms of the (forward) influence that one region exerts on the other regions, and the SMA exhibited the strongest influence of this type (Figure 4). Similarly, the (backward) influence that other regions exhibit on a particular region was strongest in the SMA. These findings indicate that the SMA may play a predominant role in efficient communication among cortical motor regions during ME in both older and young adults. The SMA has been reported to collaborate with the M1 and PMC during movement execution and the regulation of motor performance (Luppino and Rizzolatti, 2000; Cauda et al., 2011). In addition, SMA was already indicated to be a crucial region in motor control by recent studies (Deshpande et al., 2009; Chen, 2011; Xu et al., 2014; Riecker et al., 2015). It is thought to play a predominant role in efficient communication between different regions during ME by the betweenness centrality of graph theory. However, these conclusions were based on studies of young subjects. In the present study, our results further indicate that the SMA plays the same important role in motor control in older subjects.

We found that the connections in the older group during ME increased compared with those in the young group during ME (Figure 4). In other words, some connections were altered due to aging. We noted that the increases were primarily the between-hemisphere connections, which included the influence from PMC.R (right premotor cortex) to M1.L (left primary motor cortex) and from PMC.R to PMC.L (left premotor cortex). The function connectivity has been found to increase with age for the motor cortex and putamen (Seidler et al., 2015). The connectivity between the motor cortex and the bilateral SMA was associated with greater grip force. In the resting state, the higher tapping frequency and grip strength were related to the stronger connection between the motor regions. These findings suggested that older adults who have stronger sensorimotor connectivity were superior to their peers in motor function. We applied the CGCA, which confirmed the stronger between-hemisphere connections within the motor network in older adults during ME, consistent with our hypothesis. Our previous results showed that bilateral brain areas require greater activation in order for adults to complete the same level of motor performance in older adults, and this greater activation may require more between-hemisphere interactions. This greater activation with age may be related to compensatory mechanisms, in that older adults need greater between-hemisphere interactions to protect against age-related decrease of motor function.

Our previous studies have indicated that older people have recruited less lateralized motor cortex than younger people during ME, and the older people lend to recruit more brain regions (Wang L. et al., 2014), particularly the ipsilateral motor cortex, to implement motor activities (Mattay et al., 2002; Ward and Frackowiak, 2003; Heuninckx et al., 2005; Riecker et al., 2006; Langan et al., 2010). In this study, the lateralized interaction in the hemisphere was also obvious in the young group; however, the hemispheric asymmetry of the interaction was not obvious in the older group. The present study further indicated that the interactions between brain area lateralization are reduced with aging.

### Effective Connections During MI

There were many effective connections in the motor network during MI in both groups, which confirmed that MI could stimulate the movement information circuit (Gao et al., 2008b; Chen et al., 2009). Many studies have shown that MI has become an effective tool for improving and characterizing brain function at different stages after stroke (Butler and Page, 2006; Bajaj et al., 2015). Our results further showed that MI could promote the interaction between brain motor regions and is helpful for the recovery of motor function.

In our study, the connectivity between PMC and M1 was found to reduce during MI in older adults. In addition, the connectivity between SMA and contralateral M1 was also found to reduce. This reduced connectivity lies mainly in the domain hemisphere. In our previous study, we found that the activated motor cortical areas became difficult in older adults during MI. The disconnection of white matter integrity with aging may affect the connection between the cerebral cortex. Decreased motor function in older people was associated with significant decrease in white matter integrity (Wu and Hallett, 2005; Zahr et al., 2009). The connectivity between PMC and ipsilateral M1 was decreased in older adults using an electrophysiological approach (Ni et al., 2015). These studies show that the effective connection within the cortical motor network possibly decreased with aging. Now, we use the GCCA method and further confirm that the causal interaction between the motor cortex is reduced with aging during MI, which was consistent with our previous hypothesis. The functional modulation of the within-domain hemisphere connection during MI may be altered with aging.

It has reported that the PMC was a crucial region in MI by the betweenness centrality of graph theory, which maybe plays a predominant role in efficient communication between different regions (Xu et al., 2014). We confirm this result in the young group by statistical analysis of the degree of brain network parameters. However, in the older group, we found that SMA may play a predominant role in efficient within-motor network communication during MI. These results showed that the role of motor areas in the motor network may change with aging and that SMA may play a significant role in information integration in older adults during MI.

## Conclusion

The present results showed that interaction within the motor network was changed with aging during task performance. The increased between-hemisphere connectivity in the older group during ME may be related to a compensatory mechanism, in that the older adults need greater between-hemisphere interactions to protect against age-related decreases in ME. The effective connectivity decreased within-domain hemisphere connectivity in the older group during MI, implying that the interaction between cortical motor neurons caused by MI was more difficult with aging. The significantly delayed BOLD signal during MI also explained the mechanism of neural degeneration in older adults. In addition, we found that SMA played a very important role within the motor network in older adults during both ME and MI. The changes in these functional brain networks with aging may reflect abnormality of motor control systems that are predictive of neurodegenerative and neurochemical changes in older adults. Longitudinal analyses will be important in establishing this relationship.

Although our findings provide better understanding of the motor network in the health of older adults, there are several limitations in this study that need to be noted. First, this study has one young group and one older group, which lack longitudinal subjects from young to old. The next study should expand the sample size, including all the subjects from young to old, and explore the age-related significant correlation between interaction within the motor network. Second, this study did not divide SMA into two parts as with PMC/M1. It might be more accurate to subdivide SMA and study the interaction between motor cortices. In addition, we only calculated the Granger causality in the time domain but did not explore the Granger causality spectrum. Thus in the following study, we would subdivide SMA into the right and the left, studying the interaction between the subcortical and the cortical regions, and trying to calculate the frequency-dependent parametric Granger causality spectra and choose the order of the optimal model by using parametric or non-parametric methods to obtain more accurate results.

## Ethics Statement

Written informed consent was obtained from each subject before the experiment and was approved by the Third Military Medical University Ethics Committee. The methods were carried out in accordance with the approved guidelines.

## Author Contributions

MQ, LW, and CL conceived and designed the experiments. YZ, JZ, PL, and LS prepared the samples and analyzed the data. RY, LW, and CL participated in interpreting and analyzing the data. LW and CL wrote the manuscript.

## Conflict of Interest

The authors declare that the research was conducted in the absence of any commercial or financial relationships that could be construed as a potential conflict of interest.
