# Corrigendum: Aging Changes Effective Connectivity of Motor Networks During Motor Execution and Motor Imagery

**DOI:** 10.3389/fnagi.2020.00187

**Published:** 2020-07-08

**Authors:** Li Wang, Ye Zhang, Jingna Zhang, Linqiong Sang, Pengyue Li, Rubing Yan, Mingguo Qiu, Chen Liu

**Affiliations:** ^1^Department of Medical Imaging, College of Biomedical Engineering, Army Medical University, Chongqing, China; ^2^Department of Rehabilitation, Southwest Hospital, Army Medical University, Chongqing, China; ^3^Department of Radiology, Southwest Hospital, Army Medical University, Chongqing, China

**Keywords:** motor network, motor execution, motor imagery, effective connectivity, Granger causality analysis

In the original article, all authors were indicated as corresponding authors. This is incorrect and only Mingguo Qiu and Chen Liu are corresponding authors.

The authors apologize for this error and state that this does not change the scientific conclusions. The original article has been updated.

